# Studies in the Medical Botany of Southern Illinois

**Published:** 1882-02

**Authors:** J. M. G. Carter


### ARTICLE IV.

### STUDIES IN THE MEDICAL BOTANY OF SOUTHERN ILLINOIS.

By J. M. G. CARTER, A.M., M.D.

### III.

Asclepiadaceæ, or Milkweed family, cannot be said to contain any very important remedies. Still, there are at least two plants of this order growing in this portion of our State which should be mentioned as possessing medicinal virtues. These are common milkweed or silkweed (Asclepias Cornuti, A. Syriaca) and pleurisy-root (A. Tuberosa). A. Cornuti or A. Syriaca is anodyne and expectorant, alterative and cathartic in its properties. It has been recommended in scrofulous affections and bites of snakes and insects. It may be used in decoction, any part of the plant being used; the dose of which is a tablespoonful *pro re nata*.

A. Tuberosa (butterfly-weed, pleurisy-root) has properties similar to the preceding—diaphoretic, anodyne, expectorant, cathartic, and slightly astringent. It is used in pleurisy, pneumonia, consumption, diarrhœa, dysentery and rheumatism. It has been used as a gentle tonic for the stomach, and has been considered of value in the exanthematous diseases. The dose of the powder is twenty grains several times a day. It may be administered in decoction or infusion, in the proportion of ℥j to Oj. Dose tablespoonful every hour or two.

The division Apetalous Exogenous plants contains some valuable remedies.

The Pokeweed family (Phytolaccaceæ) is represented in our section by Phytolacca Decandra (common pokeweed). This plant, called pokeweed, pigeon-berry and garget, is not confined to low grounds, as is sometimes said, but may grow anywhere if the soil is rich. Its action is emetic and cathartic in large doses. It is said to have some narcotic properties. Its substitution for ipecac cannot be recommended. It has been used internally in chronic rheumatism and granular conjunctivitis with good results. It has been used locally in inflamed mammæ, and in certain cutaneous diseases. I have seen the infusion so used in scabies with apparent success. The infusion has also been recommended in piles. "An ointment, prepared by mixing an ounce of the powdered root or leaves with half a pound of lard, has been successfully used in psora, tinea capitis, and other cutaneous diseases." A tincture may be used in rheumatism, in teaspoonful doses thrice daily. The powder may be used in five-grain doses as an alterative, and in twenty-grain doses as an emetic.

Of the Buckwheat family (Polygonaceæ), we have a representative in Polygonum Hydropiper (common smartweed). This plant is little used in this region, because of its irritating properties; but it is said to have diuretic and other valuable medicinal virtues. It has been used in amenorrhœa and other uterine troubles with asserted beneficial results. The leaves may be used in powder (gr. iij to xx), infusion or decoction (℥j to Oj), the dose of which is a tablespoonful thrice daily.

Several varieties of Dock, all belonging to this family, are found in Southern Illinois. Among these may be mentioned yellow or curled dock (Rumex Crispus), pale dock (R. Brittannicus), and bitter dock (R. Obtusifolius). R. acetosellus, or common sheep-sorrel, is very abundant, and when fresh is a pleasant refrigerant in fevers. The Docks are astringent, tonic, alterative, and some of them slightly laxative — resembling rhubarb.

We have an abundance of Sassafras, a tree of the Laurel family (Lauraceæ). The Sassafras Officinale, or Laurus Sassafras, is used with us more as a demulcent and diaphoretic than as an astringent. It is a very common remedy in colds, in the form of hot tea, taken at bedtime. The bark of the root, the part generally used, has been supposed to have stimulant properties. "The complaints for which it has been particularly recommended are chronic rheumatism, cutaneous diseases, and scorbutic and syphilitic affections." The oil may be given; or, which is more common, the tea or infusion *ad libitum.*

The Mistletoe family (Loranthaceæ) is represented, in our woods, by Phoradendron Flavexeus (American Mistletoe). This poisonous plant has not been used as a medicine, so far as I am aware, but would produce results, probably, similar to European mistletoe, which was once considered to be a powerful antispasmodic, and a valuable remedy in palsy, epilepsy, and other nervous diseases.

Ulmaceæ (Elm family). I think Dr. Gray, in his Manual of Botany (Ed. of 1867), has confounded two varieties of elm under the name "Ulmus Fulva." He calls this tree "Slippery or Red Elm," and says that it has a "tough, reddish wood, and a very mucilaginous inner bark." The slippery elm of this region has a whitish, brittle, mucilaginous inner bark ; the wood near the bark being also whitish, but reddish within, and easily split. What we call red elm has a tough, reddish bark, used largely for bottoming chairs. It has an astringent taste. The reddish wood is very tough, it being scarcely possible to split it. We do not use the bark of the red elm medicinally, but the slippery elm bark is freely used as a demulcent. It often has an excellent effect in nausea and vomiting, acting as an anti-emetic. I have found frequently that the water of the bark would quiet an irritable stomach which morphine, calomel, bismuth and oxalate of cereum had failed to relieve.

The Bread Fruit and Fig family (Artocarpeæ). There is one representative of this, growing chiefly in old fields—that is the Morus Rubra (Red Mulberry). This berry is a laxative, and the juice is grateful in fevers. In the form of syrup it is valuable as a gargle in sore throat.

Urtica Dioica, or common nettle of the Nettle family (Urticeæ) grows abundantly in Southern Illinois in lanes and by roadsides. I have never made any use of this remedy, but it has been supposed to be diuretic and astringent, and has been employed in diseases where these properties might accomplish good, as in kidney diseases, hæmorrhoids, etc. Uterine hæmorrhage has been successfully treated with a decoction of the plant (℥j to Oj) in teacupful doses *pro re nata.* It has been used with asserted success in consumption, jaundice, worms, and locally in palsy. The young shoots are sometimes boiled and eaten by the people for scurvy.

Cannabis Sativa, or C. Americana (Family Cannabineæ) is found growing in waste and cultivated grounds. This may be employed in the form of the officinal preparations, or the flowering tops may be used similarly to hops as an anodyne poultice. It is of especial value in neuralgia and rheumatism. This plant has the same virtues as C. Indica, so fully described in text-books.

The four preceding families belong to the natural order Urticaceæ, of which they are sub-orders. To the same natural order (Urticaceæ), and the preceding sub-order (Cannabineæ), belongs Humulus Lupulus (Common Hop). Although Hop is indigenous to this part of the country, our chief supply is obtained from gardens, where it is cultivated. It is used very largely in domestic and professional practice as an anodyne, in the form of poultices. In severe headaches I very often secure relief by directing a hop pillow to be placed under the head. Restlessness may be controlled in the same way. Humulus has some value as a tonic in dyspepsia and intermittents. For its tonic effect, hop tea may be used. The remedy is useful in nervous tremors, wakefulness, and delirium tremens of drunkards.

Order Juglandaceæ (Walnut family). To this family belong the Walnuts (black and white), Juglans Cinerea (Butternut, White Walnut) and J. Nigra (Black Walnut). Both grow in our section of the State, the latter very abundantly. The leaves are valuable in scrofula. While J. Cinerea is the only officinal variety, J. Nigra is very likely quite as reliable a medicine.

I have frequently observed the laxative effect of the kernel of the black walnut. The inner bark of the root of either variety may be used in decoction for the same purposes as the leaves or oil. In large doses any of these parts may be used for catharsis; the mildness of the action resembles that of rhubarb.

Carya (Hickory), another member of this family, is represented by several varieties in our vicinity. I have never used the bark or leaves of this tree, nor indeed any other part, in medicine; but I have seen it used by the common people as a tonic, astringent and detergent. Those who have used it have faith in its virtues. The leaves and inner bark, probably that of the root, are most valuable. As a tonic and astringent, they are used in the form of decoction; as a detergent, as decoction or poultice. The C. Sulcata (Western Shell-bark Hickory) is the only variety I have known used; but the following varieties are found here, and are perhaps just as valuable as remedial agents:

C. Olivæformis (Pecan-nut); C. Alba (Shell-bark Hickory); C. porcina (Pig-nut); C. Amara (Bitter-nut).

Order Cupuliferæ (Oak family). The inner bark of any of the oaks may be used for medicinal purposes. There are a great many varieties of oak (Quercus) in Southern Illinois, several varieties of which I have myself examined. I have been informed that there are more than twenty varieties of the white oak. I shall name only a few of the best known varieties. They are as follows: Quercus Alba (White-oak); Q. Obtusiloba (Post-oak); Q. Macrocarpa (Bur-oak); Q. Bicolor (Swamp White-oak); Q. Humilis (Chinquapin-oak); Q. Imbricaria (Shingle-oak); Q. Aquatica (Water-oak); Q. Nigra (Black-jack); Q. Falcata (Spanish-oak); Q. Coccinea (Scarlet-oak); Q. Tinctoria (Black-oak); Q. Rubra (Red-oak); and Q. Palustris (Pin-oak).

While the bark of any of these may be used, and many of them are used by the people, only Q. Alba and Q. Tinctoria are officinal. These have both tonic and astringent properties, and may be used in either indication. The decoction is frequently recommended as a bath for debilitated children, with a view of toning up the skin and strengthening the system. The chief use I have made of oak bark is as an astringent in hæmorrhoids. I have used it where ordinary remedies had accomplished little good, and have been very much pleased with the results. I direct that a strong decoction or infusion be made, and that the part be bathed three or four times a day. Usually only a few days are required to produce a cure in any case that can be relieved without an operation.

The Willow family (Salicaceæ) is represented by several varieties in Southern Illinois, the most important of which are Salix Nigra (Black Willow), and S. Babylonica (Weeping Willow). The bark of these trees contains the active principle, salicin, from which salicylic acid may be obtained. A decoction of the bark is a good remedy in malarial fevers and rheumatism. It has tonic and astringent properties, and may be used as a substitute for Peruvian bark. It has the antiperiodic effect of quinine, and has been used with success in intermittents; but its power is inferior to that of the sulphate of quinia. "The decoction of willow has been found beneficial as an external application to foul and indolent ulcers." The powder or decoction of willow bark may be used in the same doses as cinchona.

Although not so prolific of medicinal plants as the class to which all the preceding remedies belong, still, the class of Monocotyledonous or Endogenous plants contains several remedial agents growing in Southern Illinois. I shall mention only a few of them.

Order Araceæ (Arum family). This family is represented by two common medicinal plants which I shall mention: Indian Turnip and Sweet Flag or Calamus.

Indian Turnip (Arisæma Triphyllum) has been used in asthma, whooping-cough, chronic catarrh, chronic rheumatism, and as an alterative and tonic in various cachexiæ in emulsion. It is considered stimulating to the skin and lungs. In the form of a syrup or honey it is valuable in aphthous sore mouth of children. The root should be partially dried before using, to expel some of the acrid properties. In the form of emulsion the root may be given in doses of gr. x to xxx, beginning with the minimum dose. The decoction may be used as a fomentation in diseases of the lungs where counter-irritation is indicated.

Sweet Flag (Acorus Calamus) is quite abundant in portions of our section. The plant, the root or rhizome of which is used, is an aromatic, and may be so used with children in colic and flatulence. It is a valuable adjuvant to cathartic medicines. It is useful in debility or torpor of the alimentary canal, and as a general systemic stimulant tonic. It may be given in the form of decoction or infusion (℥j to Oj, aq. bul.), the dose of which is a wineglassful. Children frequently like to chew the root. It is supposed that this plant was known to the ancient Greeks under the name acorow.

Cypripedium, of the Orchis family (Orchidaceæ), is represented in this vicinity by several varieties, three of which, C. Candidum, C. Parviflorum, C. Pubescens, are used as medicinal agents. All these, called White Lady's Slipper, Small Yellow Lady's Slipper and Large Yellow Lady's Slipper, respectively, are perhaps of equal value. The rhizome is the part used. Cypripedium is a mild stimulant to the nervous system, being valuable in the treatment of hysteria, hypochondriasis, neuralgia, etc. It may be given in the form of powder (gr. fifteen) tincture or infusion.

Iris family (Iridaceæ). Iris Versicolor (Larger Blue Flag) grows quite plentifully in this portion of the State. The rhizome is the part used. The powdered root, if kept in bottles protected from the air, may retain its virtues for several months or a year, perhaps longer. Its medical properties are cathartic, diuretic and emetic. It is useful in dropsy. It may be used in powder, decoction, infusion or tinctnre. The dose of the powder is gr. iv to xx.

This is only a moiety of the medicinal plants of Southern Illinois, but perhaps the most important are included in these brief studies.

